# Microalgal—bacterial interactions: Research trend and updated review

**DOI:** 10.1016/j.heliyon.2024.e35324

**Published:** 2024-07-26

**Authors:** Muhammad Iqhrammullah, Williams Chiari, Syihaabul Hudaa, Irhamni Irhamni, Fahrurrozi, Said Ali Akbar

**Affiliations:** a Research Center for Marine and Land Bioindustry National Research and Innovation Agency (BRIN), North Lombok, 83756, Indonesia; b Postgraduate Program of Public Health, Universitas Muhammadiyah Aceh, Banda Aceh, 23123, Indonesia; c Division of Mathematical and Physical Sciences, Graduate School of Natural Science and Technology, Kanazawa University, Kanazawa, 920-1192, Japan; d Department of Management, Institut Teknologi dan Bisnis Ahmad Dahlan Jakarta, Banten, 15419, Indonesia; e Department of Environmental Engineering, Faculty of Infrastructure and Regional Technology, Institut Teknologi Sumatera, Lampung Selatan, 35365, Indonesia; f Department of Environmental Engineering, Faculty of Engineering, Universitas Serambi Mekkah, Banda Aceh, 23245, Indonesia; g Department of Aquaculture, Faculty of Marine and Fisheries, Universitas Syiah Kuala, Banda Aceh, 23111, Indonesia

**Keywords:** Microalgae, Bacteria, Bibliometric, Algacidal, Flocculation

## Abstract

Microalgae are being recognized as the key contributor to sustainability in many sectors, starting from energy up to food industries. The microorganism has also been utilized as environmental remediator, capable of converting organic compounds into economically valuable biomass. To optimize the use of microalgae in these sectors, researchers have explored various approaches, of which is the use of bacteria. The interaction between bacteria and microalgae can potentially be harnessed, but its complexity requires extensive research. Herein, we present the bibliometric analysis on microalgal-bacterial interactions. The metadata of published literature was collected through Scopus database on August 4, 2023. The downloaded.csv file was uploaded to VOSViewer and biblioshiny for network visualization. We found that the research has gained a lot of attention from researchers since 2012 with an exponential increase of the publication number. The United States and China are leading the research with a strong collaboration. Based on the research sub-topic clusters, the interaction is mostly studied for wastewater treatment, biomass production, and algal bloom control. Updated reviews on this topic reveal that researchers are now focus on optimizing the efficacy of microalgae-bacteria system, investigating the modes of actions, and identifying challenges in its real-world implementation. The microalgal-bacterial interaction is a promising approach for microalgae utilization in wastewater treatment, biomass production, and algal bloom control.

## Highlights

• Microalgal-bacterial research has reached 2357 publications (90.4 % original articles).
• “Agricultural and Biological Sciences” is the major subject of this research topic.
• China and USA are dominating the research and publication.
• The interaction is mostly studied for wastewater treatment, biomass production, and algal bloom control.

## 1 Introduction

Microalgae has been acknowledged as the key to many sustainable productions, including energy, feedstock, foods, and pharmaceuticals. A review article published in ‘Renewable and Sustainable Energy Reviews’, in 2012, calls microalgae as the solution for world's ‘macroenergy’ [1]. *Chlorella* sp. is an example of microalgae that has been widely known to produce high fatty acids, hence high biodiesel output [2]. In addition to its importance in energy sector, microalgae have been widely used for bioremediation [3]. In fact, organic wastewater can be used as the nutrient source to cultivate microalgae for biofuel productions [4]. In aquaculture sector, not only does it act as a feedstock, microalgae also increase the immunity of the animal [5]. Microalgae, in the form of dried powder or its raw extract, could modulate immune response in vitro or in vivo [5]. With these advantages, it is no wonder that microalgae industry has been blooming in many countries, including in Malaysia, Belgium, Germany, France, Italy, India, the United States, and China [[6], [7], [8]]. The global market for microalgae was recorded at USD 3.4 billion in 2020, and estimated to grow up to USD 4.6 billion in 2027 [7].

However, there are several challenges in realizing sustainable microalgae cultivation. For example, harvesting microalgal biomass requires high amount of energy and water [[9], [10]]. Furthermore, microalgal species of interest may also be prone to toxic contaminants, such as polycyclic aromatic hydrocarbon and heavy metals [11,12], requiring intense water quality control and purification. To minimize and overcome these problems, there have been several reports suggesting the use of bacteria as eco-friendly, cheap, and non-toxic solutions. Introducing flocking bacteria to the microalgal culture could increase the biomass production yield [13]. Additionally, the formation of microalgal-bacterial flocks could increase the pollutant uptake [14]. Moreover, bacteria could provide a protective effect against toxic heavy metals by entraping the contaminants (probably through exudate formation) which effectively hinders their absorption into microalgal cells [15]. However, most of the bacteria exhibit algicidal activities which limit the water recycling process. Indeed these activities can be utilized to control harmful algal bloom [16]. Despite having a lot of potential, the interaction between microalgae and bacteria is a complex research subject. Therefore, this review informs readers on the research trend and landscape of microalgal-bacterial interactions to illustrate the current progress of this topic and help them designing future research.

To capture the research landscape of a new research topic, bibliometric approach can be employed. Our team has used the same approach to study the research trend of coronavirus disease 2019 (COVID-19) which is relatively a new topic, including those related to essential oil and polymeric materials [17,18]. In microalgae-related topics, this approach has also been used by different research groups [19,20]. By visualizing the co-occurring keywords of published literature, research trends can be observed through cluster formation. In this study, we intend to seek answers to the following research questions: (1) How was the early and late progress of ‘microalgal—bacterial interactions’ topic? (2) What are the main focuses of researchers when studying microalgal—bacterial interactions? How was the collaborative research landscape in this topic?

## 2 Methods

This study utilized Scopus database to retrieve papers related to the microalgal-bacterial interaction, where the search was performed on August 4, 2023. The keywords used in the Scopus database search were the following: “Microalga* OR Chlorella OR "Green Algae" OR Coccolithophores OR Diatom OR Phytoplankton OR Spirulina OR Dunaliella OR Nannochloropsis OR Phaedactylum OR Dinoflagellate) AND (Bacteria* OR Bacterium OR Microbe* OR Microbial OR Bacillus OR Rhizobium OR Escherichia”. No time span and language restrictions were applied in the database search. The exported data was then crosschecked to ensure there is no potential of disambiguation case such as multiple papers of same name or papers of unrelated to the study. The retrieved database was exported as CSV (.csv) file, and further used for network visualization on VosViewer 1.6.17 or Biblioshiny [21].

## 3 Results

### 3.1 Characteristics of identified papers on microalgal-bacterial interaction

A total of 2357 papers consisted of original articles (*n* = 2130, 90.4 %), review articles (*n* = 108, 4.6 %) and other documents (*n* = 119, 5 %) studying microalgal-bacterial interaction were retrieved via Scopus database. There was a significant increase of published papers in the last 10 years (2013–2023), where 1514 papers (64.23 %) were published during this period, indicating the rise in microalgae-bacteria study (*n* > 50 since 2012, Fig. 1).

**Fig. 1:**
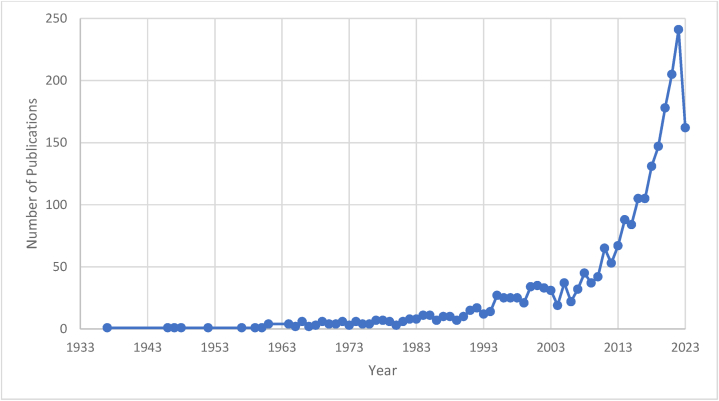
Publication trend of microalgal-bacterial interaction studies.

Agricultural and Biological Sciences (*n* = 1080, 45.8 %), Environmental Science (*n* = 928, 39.3 %), Biochemistry, Genetics and Molecular Biology (*n* = 446, 18.7 %) and Immunology and Microbiology (*n* = 445, 18.7 %) were the most studied subject areas (Table 1). The studies were published in 708 journals, where only 7 journals publishing more than 40 studies in this topic (Table S1). These 7 journals (0.98 % of total journals) collectively published 422 papers (17.89 % of total papers). Bioresource Technology (*n* = 121, 5.13 %) published the highest number of papers, followed by Algal Research (*n* = 69, 2.92 %) and Aquatic Microbial Ecology (*n* = 62, 2.63 %). These three were also the only journals publishing more than 50 papers, contributing to 10.69 % of total published papers (Table S1).

**Table 1:** Top 10 subject areas related to microalgal-bacterial interaction studies.

#	Subject Area	Number of Papers
1	Agricultural and Biological Sciences	1080
2	Environmental Science	928
3	Biochemistry, Genetics and Molecular Biology	446
4	Immunology and Microbiology	445
5	Chemical Engineering	317
6	Earth and Planetary Sciences	253
7	Energy	236
8	Medicine	196
9	Engineering	189
10	Chemistry	130

#### 3.1.1 Top 10 authors, organizations, countries, and funding sources

**Fig. 2:**
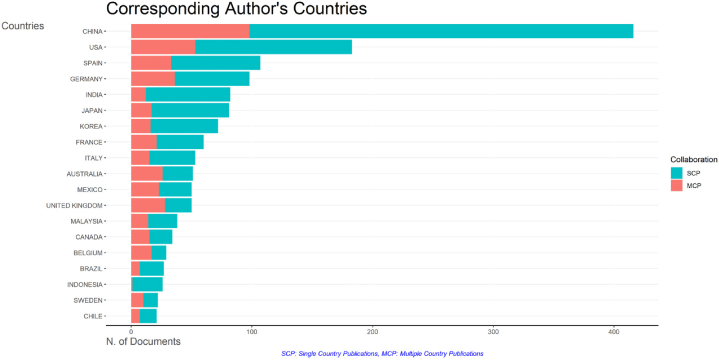
Top countries publishing microalgal-bacterial interaction research based on the corresponding authors' countries. SCP, single country publication; MCP, multiple country publication.

The most prolific author as per the data retrieval date was Ji, B. (n = 35, 1.48 %), followed by Bashan, Y. (*n* = 20, 0.84 %) and Grossart, H.P. (*n* = 19, 0.8 %) (Table S2). In terms of the most productive organizations, Chinese Academy of Sciences (n = 75, 3.18 %), CNRS Centre National de la Recherche Scientifique (*n* = 68, 2.88 %) and Ministry of Education China (*n* = 58, 2.46 %) were the top three organizations with over 50 publication records (Table S3). China (*n* = 475, 20.15 %) and United States (*n* = 439, 18.62 %) were the top publishing countries with high citation proportions (Table S4). When the analysis was carried out only on the corresponding author's countries, the two countries remained in the highest position (Fig. 2). Finally, the funding source that supported the highest number of studies was National Natural Science Foundation of China (*n* = 271, 11.49 %), followed by National Science Foundation (*n* = 91, 3.86 %) and National Key Research and Development Program of China (*n* = 58, 2.46 %) (Table 2). Deutsche Forschungsgemeinschaft (Foundation) was the only non-governmental funding source reaching the top ten list (Table 2).

**Table 2:** Top 10 funding sources for microalgal-bacterial interactions.

No	Funding Source	Status	Number of papers
1	National Natural Science Foundation of China	Government	271
2	National Science Foundation	Government	91
3	National Key Research and Development Program of China	Government	58
4	Japan Society for the Promotion of Science	Government	39
5	European Regional Development Fund	European Union	36
6	Fundamental Research Funds for the Central Universities	Government	33
7	National Research Foundation of Korea	Government	32
8	Ministerio de Economía y Competitividad	Government	32
9	Deutsche Forschungsgemeinschaft	Foundation	32
10	Consejo Nacional de Ciencia y Tecnología	Government	29

#### 3.1.2 Top 10 microalgal-bacterial interaction articles based on citation

There was a total of 72260 citations for 2357 papers, averaged 30.67 citations per paper and 840.23 citations per year (1937–2022). The most cited paper was “Master recyclers: features and functions of bacteria associated with phytoplankton blooms” authored by Alison et al. in 2014, cited in 703 papers. On the second position, “Interaction and signalling between a cosmopolitan phytoplankton and associated bacteria” authored by Amin et al. in 2015 received 628 citations. Only six papers were cited more than 500 times, with 4 of them published in the 21st century. The data of the top 10 most cited papers have been presented (Table 3).

**Table 3:** Top 10 most cited published article reporting on microalgal-bacterial interactions.

Rank	Title	Author(s)	Year of Publication	Times of Citations	Ref.
1	Master recyclers: features and functions of bacteria associated with phytoplankton blooms.	Alison et al.	2014	703	[22]
2	Interaction and signalling between a cosmopolitan phytoplankton and associated bacteria	Amin et al.	2015	628	[23]
3	Interactions between diatoms and bacteria	Amin et al.	2012	625	[24]
4	Respiration rates in bacteria exceed phytoplankton production in unproductive aquatic systems	Del Giorgio et al.	1997	587	[25]
5	The production of dissolved organic matter by phytoplankton and its importance to bacteria: Patterns across marine and freshwater systems	Baines et al.	1991	570	[26]
6	Dynamics of bacterial community composition and activity during a mesocosm diatom bloom	Riemann et al.	2000	519	[27]
7	Zooming in on the phycosphere: The ecological interface for phytoplankton-bacteria relationships	Seymour et al.	2017	486	[28]
8	Accelerated dissolution of diatom silica by marine bacterial assemblages	Bidle, K.D. & Azam, F.	1999	431	[29]
9	Marine diatom species harbour distinct bacterial communities	Grossart et al.	2005	411	[30]
10	Dual purpose microalgae-bacteria-based systems that treat wastewater and produce biodiesel and chemical products within a Biorefinery	Olguín, E.J.	2012	357	[31]

#### 3.1.3 Results from co-authorship networking analysis

The relationship between authors (including the affiliated institutions and countries) could be observed through network analysis on the co-authored papers. The analysis could suggest the collaboration trends along with the identification of prominent authors, organizations, and countries focusing their work on microalgal-bacterial interaction. The visualization maps were generated using the VosViewer software, which showed nodes of various sizes and colors representing the author, organization, or country in their own clusters. These nodes were also connected by a line—Link Strength (LS), where the LS reflects the number of co-authored papers and collaboration strength among two authors/organizations/countries. Every link connected to a certain node were summed and expressed as Total Link Strength (TLS), which was an indication for the strength of the relationship among authors, organizations, or countries [32].

The authors’ co-citation was conducted with a minimum number of citations of an author was set to 200. Out of 130786 cited authors, 101 met the aforementioned criteria. The network visualization and density visualization maps were presented in Fig. S1. Azam, F. was the author holding the highest number of citations (citations: 1165, TLS: 25353), and were followed closely by Liu, Y. (citations: 882, TLS: 37110), Bashan, Y. (citations: 867, TLS: 20083) and Li, Y. (citations: 753, TLS: 23114). It is interesting to note that the difference in number of Total Link Strength (TLS) did not determine the number of citations since it represented how often an author was co-cited with other authors on the same paper. Organization-based co-authorship analysis was performed on 5515 identified organizations, with a minimum threshold of 5 papers per organization and no minimum citation requirement. Twenty-two organizations met the thresholds, with only 5 organizations were found to be co-linked with each other (Fig. S2). School of Civil and Environmental Engineering, Nanyang Technological University Singapore (Documents = 13, citations = 482, TLS = 26) and Advanced Environmental Biotechnology Centre, Nanyang Technological University Singapore (Documents = 11, citations = 467, TLS = 25) were the leading organizations. The lack of connected nodes as shown in the visualization map indicated the current lack of collaboration among organizations, with only the aforementioned organizations having intense collaborations (LS = 11) (Fig. S2).

Lastly, country-based co-authorship analysis was carried out on 100 identified countries, where a minimum threshold of 10 papers per country and at least 5 citations per country was applied. Of 100 countries, 48 met the threshold (Fig. S3). The top three countries ordered by number of documents were China (documents = 475, citations = 8062, TLS = 190), United States (documents = 439, citations = 20215, TLS = 310) and Germany (documents = 169, citations = 6783, TLS = 158). There were only four countries publishing more than 150 papers, including the previously mentioned top three and Japan. In terms of collaboration, United States & China had the highest number of collaborations (LS = 52), followed by United States & Germany (LS = 28) and United States & United Kingdom (LS = 23) (Fig. S3). This data is also supported by the number of multiple country publication presented in Fig. 2.

**Fig. 3:**
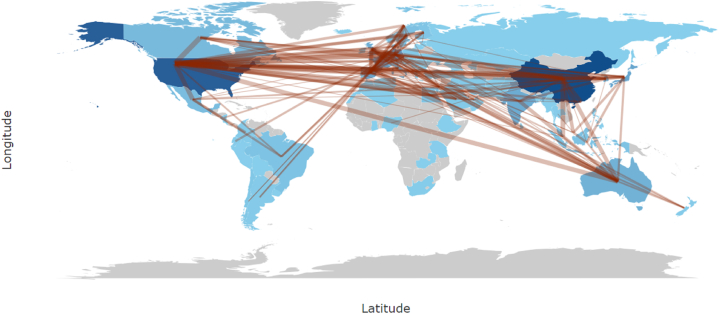
Collaboration map of countries reporting microalgal-bacterial interactions. From light to dark green indicates the increasing number of publications, while grey color indicates the absence of publication. Red lines indicate the collaboration between countries. (For interpretation of the references to color in this figure legend, the reader is referred to the Web version of this article.)

The collaboration network was also visualized as a collaboration map, presented in Fig. 3. This corroborates the findings that a strong collaboration has been established between USA and China in microalgal-bacterial interaction topic. Some countries such as Australia, United Kingdom, and Germany might not having publications as high as USA or China, but their collaboration networks are among the pronounced ones. Some low and middle-low-income countries have not published any Scopus-indexed research paper about microalgal-bacterial interactions, suggesting the presence of disparity.

### 3.2 Keyword Co-occurrence analysis

A keyword co-occurrence analysis was conducted in this study using VosViewer software to find out how frequent the connected terms or words used in papers related to a specific field of research and identify the developing trend of the research focus, generally grouping the keywords into clusters. Each node in the visualization map indicates the keyword, while the link line connecting the nodes indicates two certain keywords’ relation and their use on a paper. Every node and link line are varied in size, which indicates the popularity of a keyword.

**Fig. 4:**
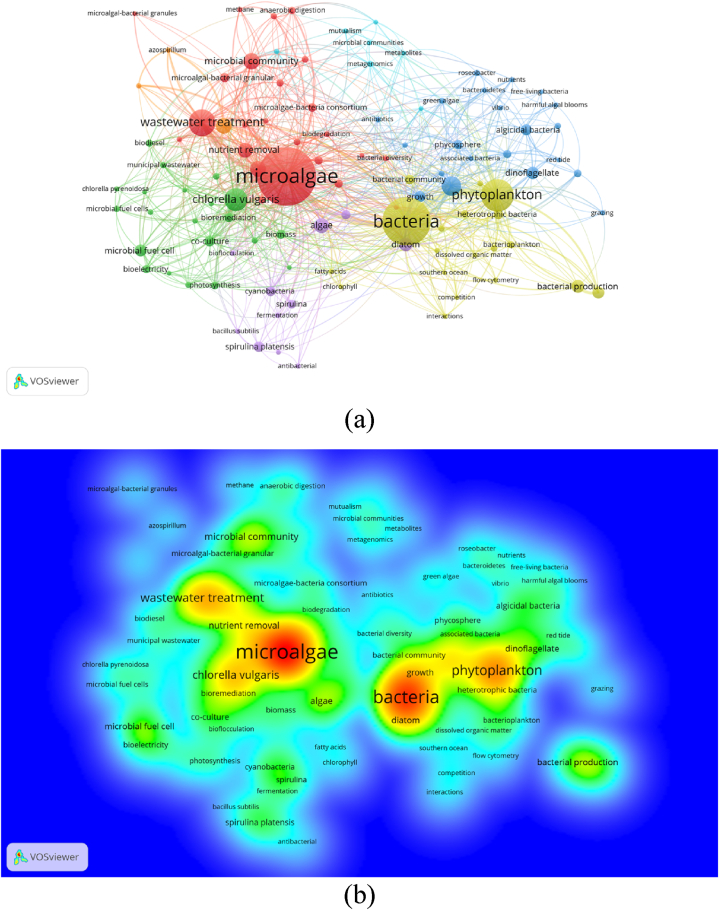
(a) Network Visualization of keywords' co-occurrences (weights: occurrences). (b) Density visualization of keywords' co-occurrences (weights: occurrences).

In this study, a total of 4841 keywords were identified with a restriction of minimum number of occurrences of a keyword was set to 9. Of 4841 keywords, 106 met the threshold. The most used authors keyword in the study on microalgal-bacteria interaction was ‘microalgae’ (occurrences = 316), followed by ‘bacteria’ (occurrences = 238), ‘phytoplankton’ (occurrences = 136) and ‘wastewater treatment’ (occurrences = 102). Only four keywords passed the 100 occurrences-mark, indicating how most papers were related to these keywords. The network visualization shown in Fig. 4a shows different colors based on the keywords' closeness to a certain research theme, which were then grouped into clusters. Following careful selection by the reviewers, at least the research can be divided into three clusters (wastewater treatment, biomass production, and algal bloom control; Table 4).

**Table 4:** Clusters of trending microalgal-bacterial research sub-topics.

Cluster (color)	Keywords of interest	Research trend
Cluster I (Red)	Wastewater, microalgal-bacterial granules, anaerobic digestion.	Microalgal-bacterial interactions for wastewater treatment
Cluster II (Green)	Biomass production, lipid, biofuel.	Microalgal-bacterial interactions for biomass production
Cluster III (Yellow)	Eutrophication, microbial loop, phytoplankton	Microalgal-bacterial interactions for algal bloom control
Cluster IV (Blue)	Algicidal bacteria, harmful algal bloom	

Density visualization of the keywords occurred in microalgal-bacterial interactions research was presented in Fig. 4b. The analysis indicated keywords in red were keywords with the highest density, followed by keywords grouped in yellow, green, cyan, and blue. The visualization suggested that ‘microalgae’ was the keyword with the highest density, followed by ‘bacteria’ and ‘phytoplankton’. Other keywords grouped in the yellow area were related to wastewater treatment and bacteria bioremediation.

## 4 Discussion

### 4.1 Research trend and landscape of microalgal-bacterial interaction

A bibliometric analysis in this study was performed based on the 2357 retrieved papers related to microalgal-bacteria interaction topics from the Scopus database. This analysis was conducted to thoroughly assess the study status in accordance to the leading authors/organizations/countries, source journals and their collaboration between one another. Bioresearch Technology published the highest number of microalgae and bacteria relation studies (*n* = 121, 5.13 % of total papers), followed by Algal Research (2.92 % of the papers). Author Ji, B. published the largest number of papers followed by Bashan, Y. In terms of organization, Chinese Academy of Science (*n* = 75, 2.95 % of total papers) and CNRS Centre National de la Recherche Scientifique (*n* = 68, 2.88 %) were leading the publication frequency. However, the leading co-authoring organizations were School of Civil and Environmental Engineering and Advanced Environmental Biotechnology Centre which were under the same institution – Nanyang Technological University Singapore.

Countries' co-authorship analysis showed that China (*n* = 475, citations = 8062), United States (*n* = 439, citations = 20215) and Germany (*n* = 169, citations = 6783) were countries with the highest number of publications. The United States and China also had the highest number of collaborations, working together on 52 occasions. China's dominance in the number of publication is largely attributed to the fact that Chinese government agencies were actively funding research in this topic. This finding is in line with a previous bibliometric study, where microalgae bio-granulation research was predominated by authors from China [19]. This report is close to the present study, since bio-granulation of microalgae can also be resulted from microalgal-bacterial interaction [14]. However, on general microalgal research topic, a published reported revealed that the United States occupied the large portion of publications [8].

### 4.2 Microalgal-bacterial interactions in wastewater treatment

Microalgae has been well known for their use in wastewater treatment, including in the removal of organic contaminants and antibiotics, and heavy metals [33]. This activity can be augmented by introducing bacterial inoculum to the bioreactor. Interaction between the two microorganisms may result in several phenomena, one of which is the formation of flocks, that is later followed by passive contaminant uptake through adsorption [33]. Nevertheless, new studies have shed light on other pathways for the contaminant removal which can be attributed to the complexity of the interaction. For example, the ammonium intake from wastewater is dependent on the pH change following the addition of microalgae [34]. Researchers have also investigated the survivability of bacterial or microalgal consortium, and found that amounts of ammonium and nitrate are the limiting factors [34].

It is noteworthy that microalgal-bacterial interaction often involves extracellular electron transfer. For instance, in ammonia removal, microalgae generate oxygen through photosynthesis, creating aerobic conditions that support ammonia-oxidizing bacteria [35]. These bacteria utilize ammonia as an electron donor, converting it to nitrite and subsequently to nitrate. Concurrently, microalgae release organic carbonaceous substances as byproducts of photosynthesis, which serve as electron donors for denitrifying bacteria [36]. This process facilitates the reduction of nitrate to nitrogen gas, completing the nitrogen cycle. In the case antibiotics biodegradation, the electron transfer from microalgae to bacteria could promote the enzymatic reactions [37]. Furthermore, microalgae provide a conducive environment for these bacteria within biofilms, enhancing the efficiency of antibiotic removal [36,37].

Several recent studies on microalgal-bacterial interaction and its use in wastewater treatment have been presented in Table 5. The technique has been reported efficient for removing various aqueous contaminants [38,39]. A study revealed that the consortia, along with the addition of Fe^3+^, could promote biodegradation of motor oil for over 99 % [32]. Emerging contaminants such as tetracycline, ciprofloxacin, and sulfadiazine (antibiotics frequently used in swine industries) could also be treated with the microalgal-bacteria consortium [40]. The microalgae mostly used for the study is *Chlorella vulgaris*, since it can be harvested for its biomass [38]. The photobioreactor can be designed with batch setting or continuous-flow setting [38,40,41].

**Table 5:** Updated reports on microalgal-bacterial interactions in wastewater treatment.

Author, Year [Ref]	Organisms		Experimental design/setting	Main findings
	Microalgae	Bacteria		
Yang et al., 2023 [34]	Chlorococcum sp. GD	Wastewater indigenous bacteria (*i.e*. *Sphaerotilus*, *Bdellovibrio*, and *Cloacibacterium*)	Exogenous Chlorococcum sp. is inculated to wastewater containing various bacteria.	- Reduced COD. - Reduced ammonium and increased nitrate with concentration dependent.
Li et al., 2023 [39]	*Chlorella vulgaris*	Acidogenic fermentation by anaerobic sludge inoculum.	Novel BACR to treat mariculture wastewater.	- High removal of COD (82.30 %), NH_3_–N (81.12 %), and total phosphorous (96.40 %).
Pi et al., 2023 [32]	*Chlorella vulgaris*	*Rhodococcus erythropolis*	MBC was added to waste motor oil TEAs (Fe^3+^ and SO_4_^2−^)	- Biodegradation of waste motor oil up to 99.2 % with Fe^3+^
Nagabalaji et al., 2023 [38]	*Chlorella vulgaris, Scenedesmus* sp.*,* and Chlorococcum sp.	Bacterial consortia grown in Nakos and Wolcott medium	MBC was added with bacterial inoculum, and further used for tannery wastewater.	- COD and TKN removals reached 89.9 % and 80.9 %, respectively.
Zambrano et al., 2023 [40]	*Scenedesmus almeriensis*	Not reported.	MBC was added to wastewater from pig slurry containing tetracycline, ciprofloxacin, and sulfadiazine.	- Removal of tetracycline, ciprofloxacin, and sulfadiazine reached 77 %, 90 %, and 60 %, respectively.
Sousa et al., 2023 [42]	*Chlorella vulgaris*	*Acidovorax facilis*	Bioremediation of methylparaben-spiked synthetic wastewater using MBC	- Methylparaben was removed (0.12 mg/L.d). - Nitrogen and phosphorous removals were lower than *C. vulgaris* alone
Xiaoyuan et al., 2023 [41]	*Chlorophyta* (not specified)	*Cyanobacteria* and *Chloroflexi* (not specified)	MBC was continuous-flow photobioreactor for municipal wastewater treatment.	- Removals of TOC, total nitrogen, and phosphorous reached 90 %, 71.4 %, and 72.6 %, respectively.

BACR, Bacteria-Algae Coupling Reactor; COD, chemical oxygen demand; MBC, microalgae-bacteria consortia; TEA, terminal electron acceptor; TKN, Total Kjeldahl Nitrogen; TOC, total organic carbon.

### 4.3 Microalgal-bacterial interactions in biomass production

During their growth, bacteria may produce extracellular polymeric substances (EPS) which act as glue to stick the microalgae together. Some of these microalgae are motile and become attracted to the bacterial EPS which subsequently turns into microalgal flocks [43,44]. The interaction between the microorganisms does not only induce the flocculation but also affect the productivity (either biomass or bioactive compounds) [[43], [44], [45]]. The coculture technique can be employed so that when the bacteria have produced sufficient EPS, the auto-flocculation will occur [43,45]. Other than relying on the bacterial EPS, bacterial dead cells can also be added to induce flocculation, where the flocculation occurs because of the electrostatic force [46].

Some updated reports on this sub-topic are presented in Table 6. The bacteria which were selected to induce the flock formation were varied, where some researchers used unspecified bacteria-enriched media [47,48]. By growing a microalgae-bacteria consortium (MBC), the harvesting efficiency could reach over 90 % [43,47]. Another option is to use bacterial cells, which can be added to the microalgae culture after being cultivated in different media [49,50]. To optimize the harvesting efficiency, augmentation by adding different species of algae can be done [48]. An economic estimate from the published report revealed that to harvest 1 kg of biomass requires only 1.35 USD [49]. However, a challenge in using bacteria in harvesting microalgae is water reusability, as the remaining bacteria in the water may have algicidal activities which can inhibit microalgal growth.

**Table 6:** Updated reports on microalgal-bacterial interactions in biomass production.

Author, Year [Ref]	Organisms		Experimental setting	Main findings
	Microalgae	Bacteria		
Devi et al., 2023 [43]	*Scenedesmus* sp.	*Limnothrix* sp.	MBC	- Higher lipid (46.2 %) - Higher efficiency (99.5 %)
Zhang et al., 2022 [44]	*Dictyosphae-rium* sp.	*Pseudomonas* sp.	MBC in swine wastewater	- Biomass (3.4–3.5 g/L) - Carbohydrate (18.4–39.3 %)
He et al., 2022 [49]	*Chlorella pyrenoidosa*	*Citrobacter* W4	Wastewater PBR + bacterial cells	- Efficiency: 87 % - Harvesting cost: $1.35/1 kg biomass
Lakshmikandan et al., 2023 [45]	*Asterococcus limneticus* WL2	*Streptomyces rosealbus* MTTC 12951	MBC	- Efficiency: 88 % - Higher biomass (24 %) - Higher lipid (22 %) - Cost: 148 % less
Nguyen et al., 2019 [47]	*Chlorella vulgaris*	Aerobic bacteria	Seafood wastewater PBR with aerobic bacteria and *Coliforms*	- Efficiency: 92 % - Dry biomass: 107.2 g/L
Siyu et al., 2022 [50]	*Spirulina platensis*	*Cobetia marina* MCCC1113	PBR + bacterial cells	- Efficiency: 94 %
Jeeraporn et al., 2022 [48]	*Scenedesmus dimorphus* and *Chlorella* sp.	Native bacteria in aquaculture wastewater	Aquaculture wastewater PBR with dual bio-augmentation	- Efficiency: 72 %

MBC, microalgae-bacteria consortia.

### 4.4 Microalgal-bacterial interactions in algal bloom control

Recent publications on microalgal-bacterial interactions in the context of algal bloom control are summarized and presented in Table 7. In controlling algal bloom, researchers rely on the effectiveness of the algicidal activities of the bacteria [[51], [52], [53]]. The bacteria release toxins that can impair the oxidative stress balance in the microalgal cells, proven by the increased activity of endogenous antioxidants [51,52]. The introduction of bacterial inoculum might alter the microorganism composition, which could devastate the microalgal population [54]. Dead bacterial cells may also act as flocculating agent and have been shown to be effective in microalgae removal, especially when combined with CaCl_2_ and FeCl_3_ [46]. In terms of efficiency, the removal could reach 75–100 % [46,51,52]. However, the removal efficiency might be lower when used in environmental settings [53]. Hence, studying the efficiency against wild microalgae is important. Additionally, other bacteria with high adaptability might outcompete the algicidal bacteria, but this usually occurs on the late stage [53]. Another consideration is the ecotoxicity of the bacterial toxins, though studies so far have shown that they are safe [55].

**Table 7:** Updated reports on microalgal-bacterial interactions in algal bloom control.

Author, Year [Ref]	Organisms		Experimental setting	Main findings
	Microalgae	Bacteria		
Furusawa and Iwamoto, 2022 [46]	*Microcystis aeruginosa*	*Aureispira* sp. CCB-QB1	Dead bacterial cells + CaCl_2_ + FeCl_3_	− 1.2 times higher removal. - Removal: 75.39 %
Zhou et al., 2023 [55]	*M. aeruginosa*	*Agrobacterium* sp	CHEC + AMP	- Removal: 85 % - Non-ecotoxic
Adi et al., 2023 [56]	*Phaeobacter inhibens*	*Gephyrocapsa huxleyi*	MBC	- Algicidal activity by denitrification
Liu et al., 2023 [51]	*M. aeruginosa*	Brevibacillus sp	PBR + bacterial cells/supernatant	- Removal: 100 % − 78.65 % increase in MDA - 3× reusability (78.65 %).
Jia et al., 2023 [52]	*M. aeruginosa*	*Paebubacillus* sp. A9	PBR + bacterial cells/supernatant	- Removal: 91 % - Increased activities of CAT and SOD
Chen et al., 2023 [54]	*M. aeruginosa*	*Pseudomonas* sp. Go58	Bacterial inoculum	- Suppresses *M. aeruginosa* - Promotes several eukaryotic bacteria
Zhuo et al., 2023 [53]	*M. aeruginosa*	*Cellvibrio* sp. G1 and *Chitinimonas* sp. G2	Bacterial broth culture + eutrophic water (wild microalgae)	- Removal: 41.3 % - Outcompeted by *Pseudomonas* sp. at the late stage.

AMP, *Agrobacterium* mucopolysaccharides; CAT, catalase; CHEC, cationic hydroxyethyl cellulose; MDA, malondialdehyde; SOD, superoxide dismutase.

### 4.5 Future research trajectories

Despite observations on microalgal-bacterial interactions have been reported since a long time ago, the increasing frequency of the publication suggests the growing trend of this research topic. With development in science and technology, more potential benefits can be drawn from the interaction between the two microorganisms. In the future, researchers may utilize gene-editing techniques to clone bacteria from specific areas and characterize their interaction with microalgae. This also means that isolation and molecular identification of bacteria are integral parts of future trajectory for microalgae research. Genetic mutations of microalgae or bacteria can also be explored as a means to optimize the interaction. For example, the technique can be used to increase microalgal growth but remain effective in forming flocks with the bacteria. Employing artificial intelligence in optimizing the interaction effectiveness, on one hand, could be a significant modality in microalgae industry development. On the other hand, fundamental research to elucidate the underlying mechanisms is still necessary especially when it implicates biomass and metabolite production, algacidal activity, and wastewater removal efficiency. In bioengineering the two microorganisms, understanding the underlying mechanisms is essential.

## 5 Conclusions

The topic of microalgal-bacterial interaction is receiving a lot of attention from researchers worldwide since 2012. At least, research in this topic can be divided into three clusters that utilizes the interaction for wastewater treatment, biomass production, and algal bloom control. Some recent studies report the utilization of this interaction to treat antibiotics contamination, though others still explore the potential on organic contaminants (COD, nitrogen, or phosphorous). The bioreactor is often designed to handle both organic contaminant removal as well as biomass production. Recent studies are investigating the mechanism behind algicidal activities of the bacteria. This research is dominated by China and United States, where the two countries have more pronounced collaboration among other countries.

## Funding

This study received no external funding.

## Data availability

The data used in this study can be accessed publicly on Scopus.

## CRediT authorship contribution statement

**Muhammad Iqhrammullah:** Writing – review & editing, Writing – original draft, Methodology, Investigation, Conceptualization. **Williams Chiari:** Writing – review & editing, Writing – original draft, Methodology, Investigation, Conceptualization. **Syihaabul Hudaa:** Writing – review & editing, Investigation. **Irhamni Irhamni:** Writing – review & editing, Validation. **Fahrurrozi:** Writing – review & editing, Supervision. **Said Ali Akbar:** Writing – review & editing, Validation, Supervision.

## Declaration of competing interest

The authors declare that they have no known competing financial interests or personal relationships that could have appeared to influence the work reported in this paper.
