# Supplementary material for: Microalgal—bacterial interactions: Research trend and updated review
Source: Heliyon. 2024 Jul 26;10(15):e35324. doi: 10.1016/j.heliyon.2024.e35324 (PMC11336587; doi:10.1016/j.heliyon.2024.e35324)
Supplement: Multimedia component 1 [file mmc1.docx]

Table S1. Top 10 journals publishing articles related to microalgal-bacterial interaction

| # | Journal | Number of Papers | Number of Citations |
| --- | --- | --- | --- |
| 1 | Bioresource Technology | 121 | 4597 |
| 2 | Algal Research | 69 | 1371 |
| 3 | Aquatic Microbial Ecology | 62 | 2433 |
| 4 | Science of The Total Environment | 45 | 1033 |
| 5 | Marine Ecology Progress Series | 43 | 2527 |
| 6 | Limnology and Oceanography | 41 | 3676 |
| 7 | Journal of Applied Phycology | 41 | 750 |
| 8 | Applied and Environmental Microbiology | 38 | 2767 |
| 9 | Microbial Ecology | 38 | 1683 |
| 10 | Frontiers in Microbiology | 38 | 1136 |

Table S2. Top 10 researchers working on microalgal-bacterial interaction studies

| No | Author | Number of Papers |
| --- | --- | --- |
| 1 | Ji, B. | 35 |
| 2 | Bashan, Y. | 20 |
| 3 | Grossart, H.P. | 19 |
| 4 | Muñoz, R. | 16 |
| 5 | Liu, Y. | 15 |
| 6 | Buitrón, G. | 14 |
| 7 | Chang, J.S. | 14 |
| 8 | Imai, I. | 13 |
| 9 | de-Bashan, L.E. | 13 |
| 10 | Gutierrez, T. | 12 |

Table S3. Top 10 organizations working on microalgal-bacterial interactions

| No | Organization | Number of Papers |
| --- | --- | --- |
| 1 | Chinese Academy of Sciences | 75 |
| 2 | CNRS Centre National de la Recherche Scientifique | 68 |
| 3 | Ministry of Education China | 58 |
| 4 | Xiamen University | 44 |
| 5 | Sorbonne Université | 37 |
| 6 | Wuhan University of Science and Technology | 35 |
| 7 | Harbin Institute of Technology | 32 |
| 8 | Universiteit Gent | 31 |
| 9 | Centro de Investigaciones Biologicas Del Noroeste | 30 |
| 10 | Scripps Institution of Oceanography | 29 |

Table S4. Top 10 countries of the authors affiliation of published articles reporting on microalgal-bacterial interactions

| No | Country | Number of Papers | Number of Citations |
| --- | --- | --- | --- |
| 1 | United States | 439 | 20215 |
| 2 | China | 475 | 8062 |
| 3 | Germany | 169 | 6783 |
| 4 | United Kingdom | 122 | 4328 |
| 5 | France | 148 | 5962 |
| 6 | Australia | 105 | 5512 |
| 7 | Spain | 147 | 5301 |
| 8 | Japan | 150 | 3289 |
| 9 | Sweden | 51 | 2039 |
| 10 | Canada | 76 | 3558 |


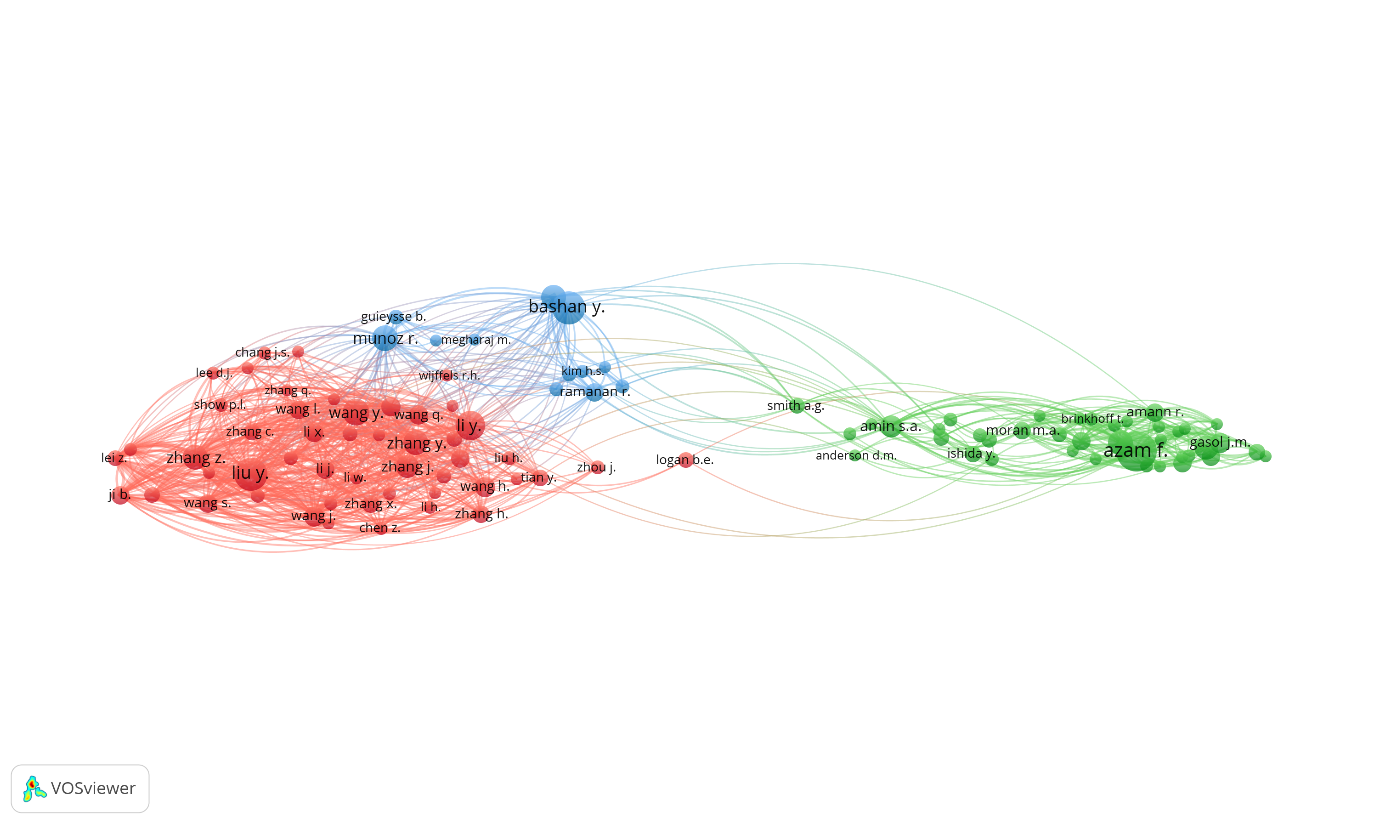


(a)


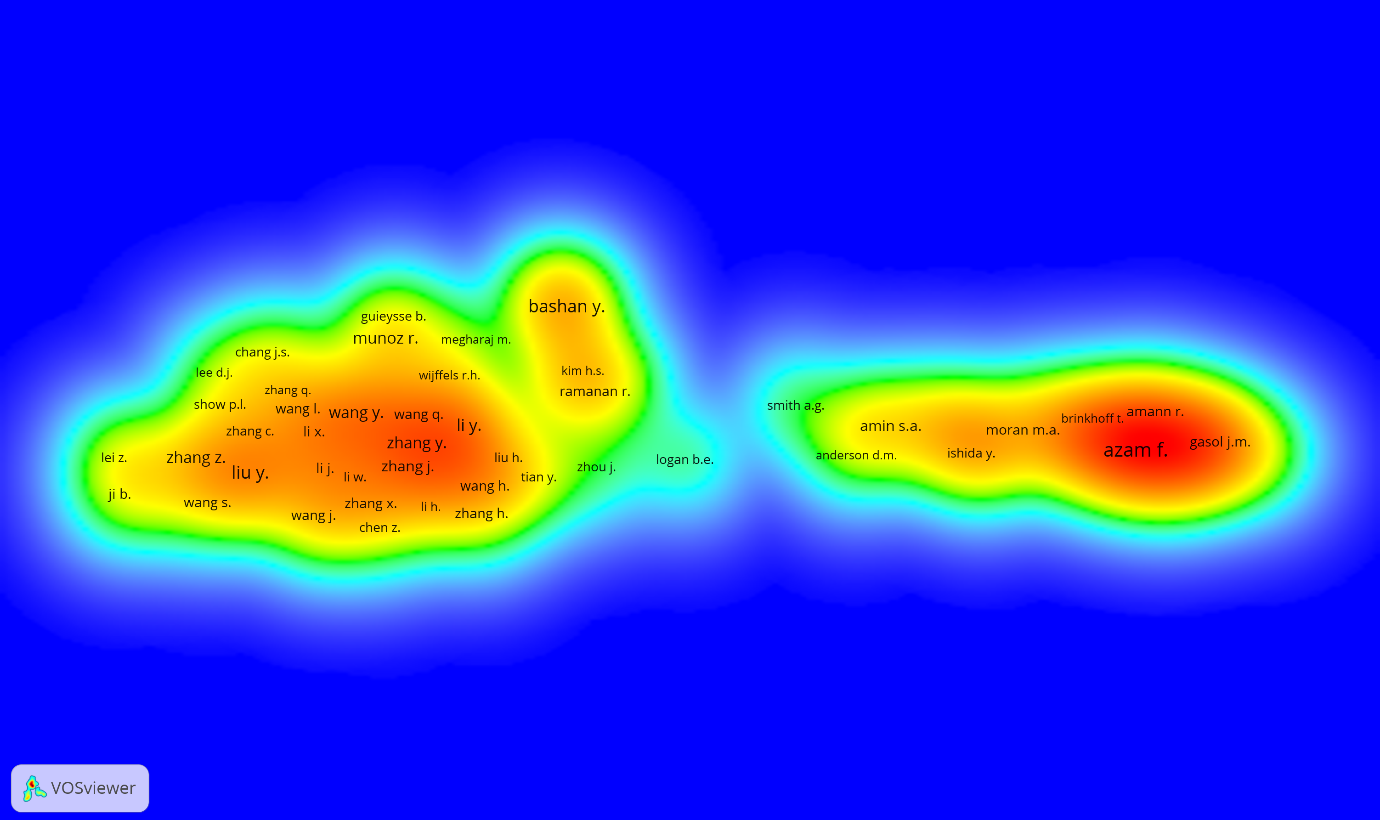


(b)

Figure S1. (a) Network Visualization of authors’ co-CITATION (weights: documents). (b) Overlay visualization of authors’ co-CITATION in 1937-2023 (weights: documents; scores: average publications per year)


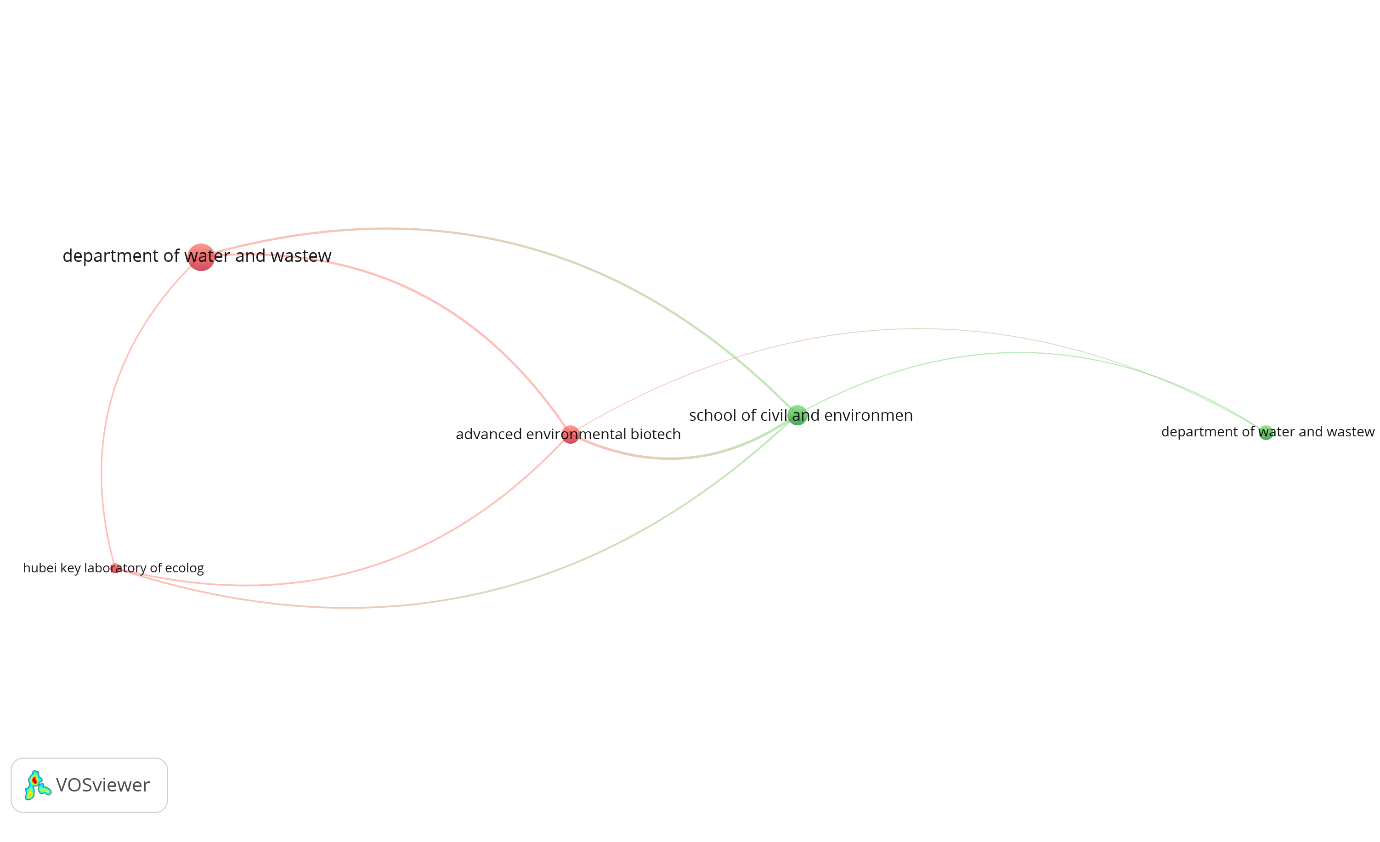


(a)


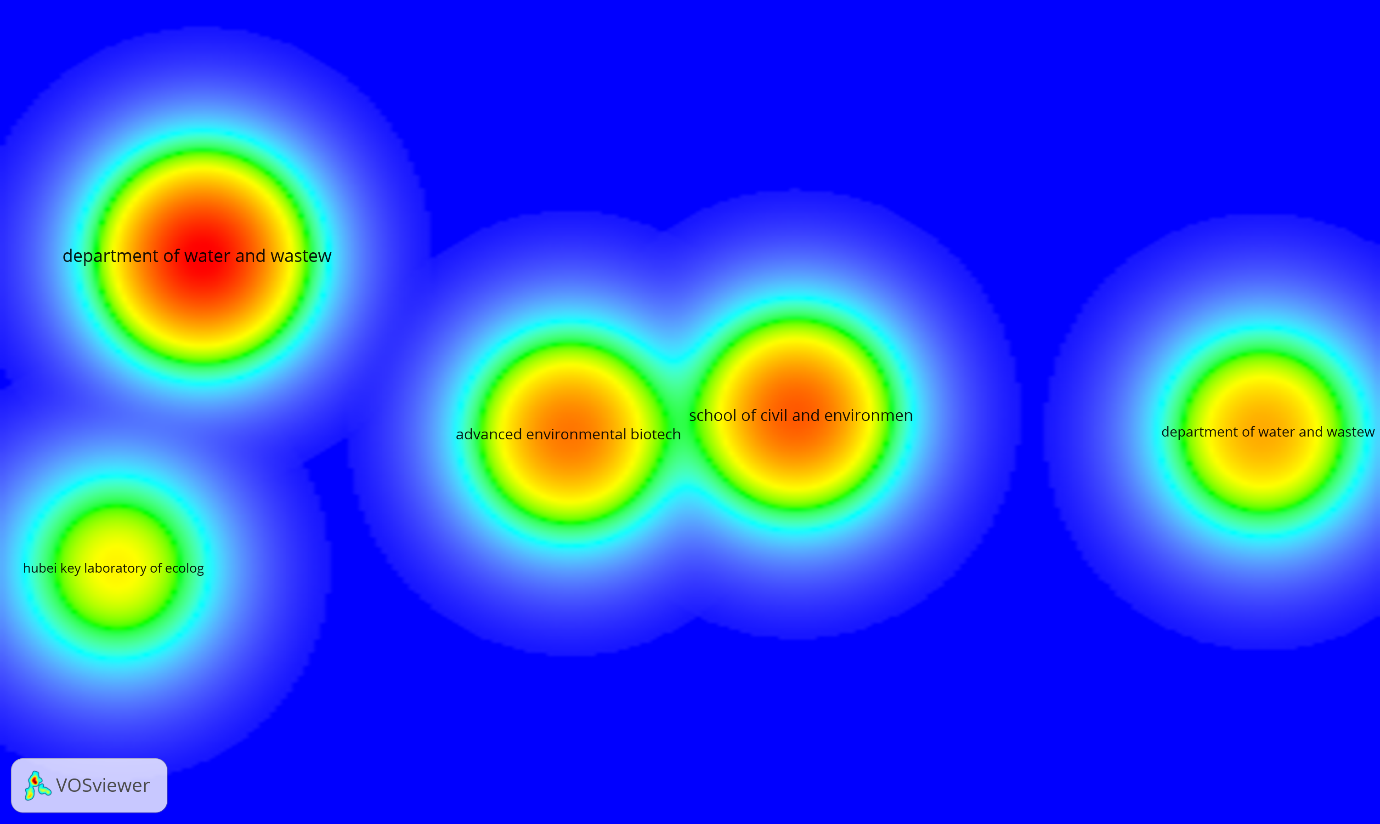


(b)

Figure S2. (a) Network Visualization of organizations’ co-authorship (weights: documents). (b) Overlay visualization of organizations’ co-authorship in 1937-2023 (weights: documents; scores: average publications per year). Only connected organizations were shown.


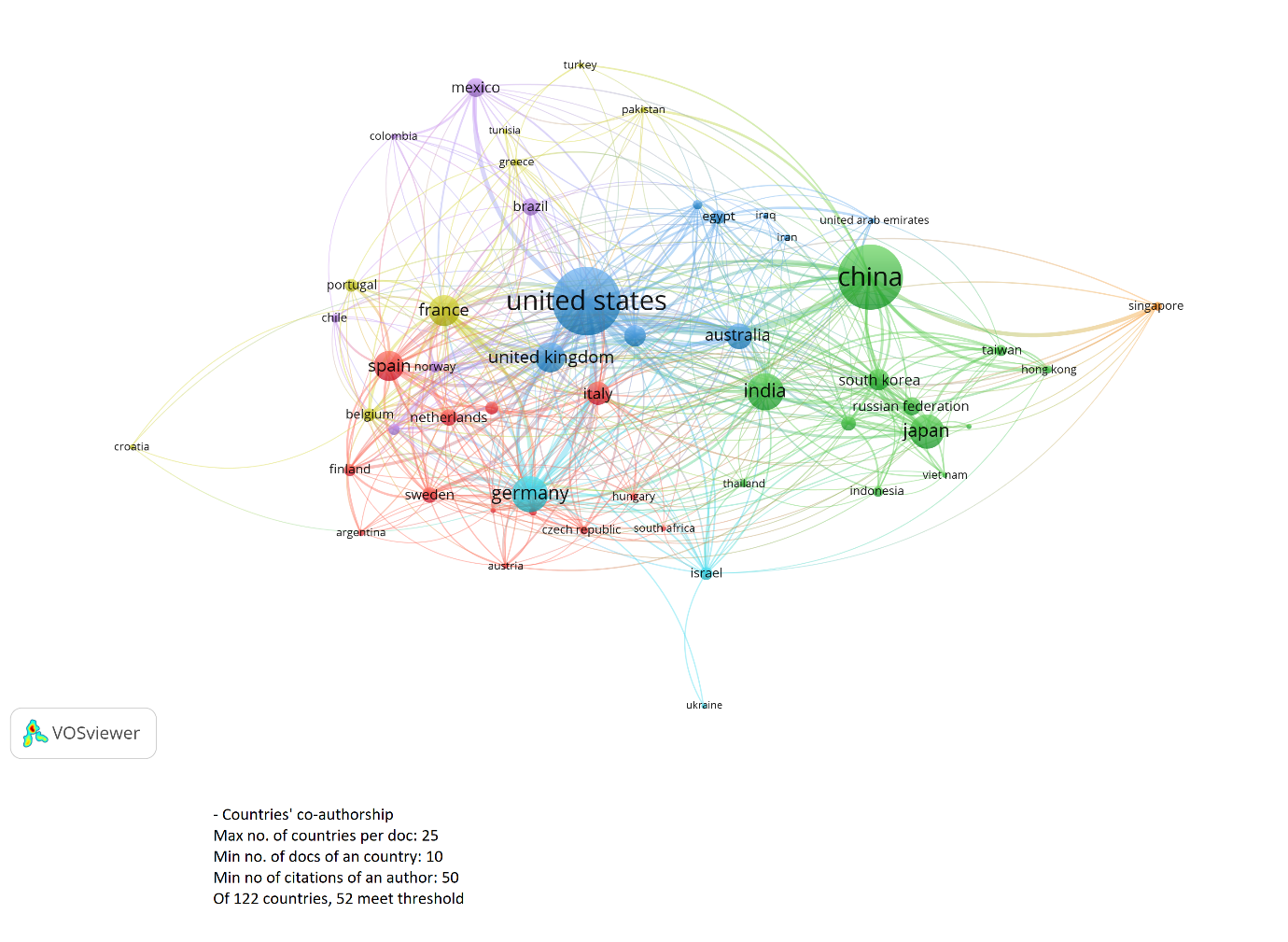


(a)


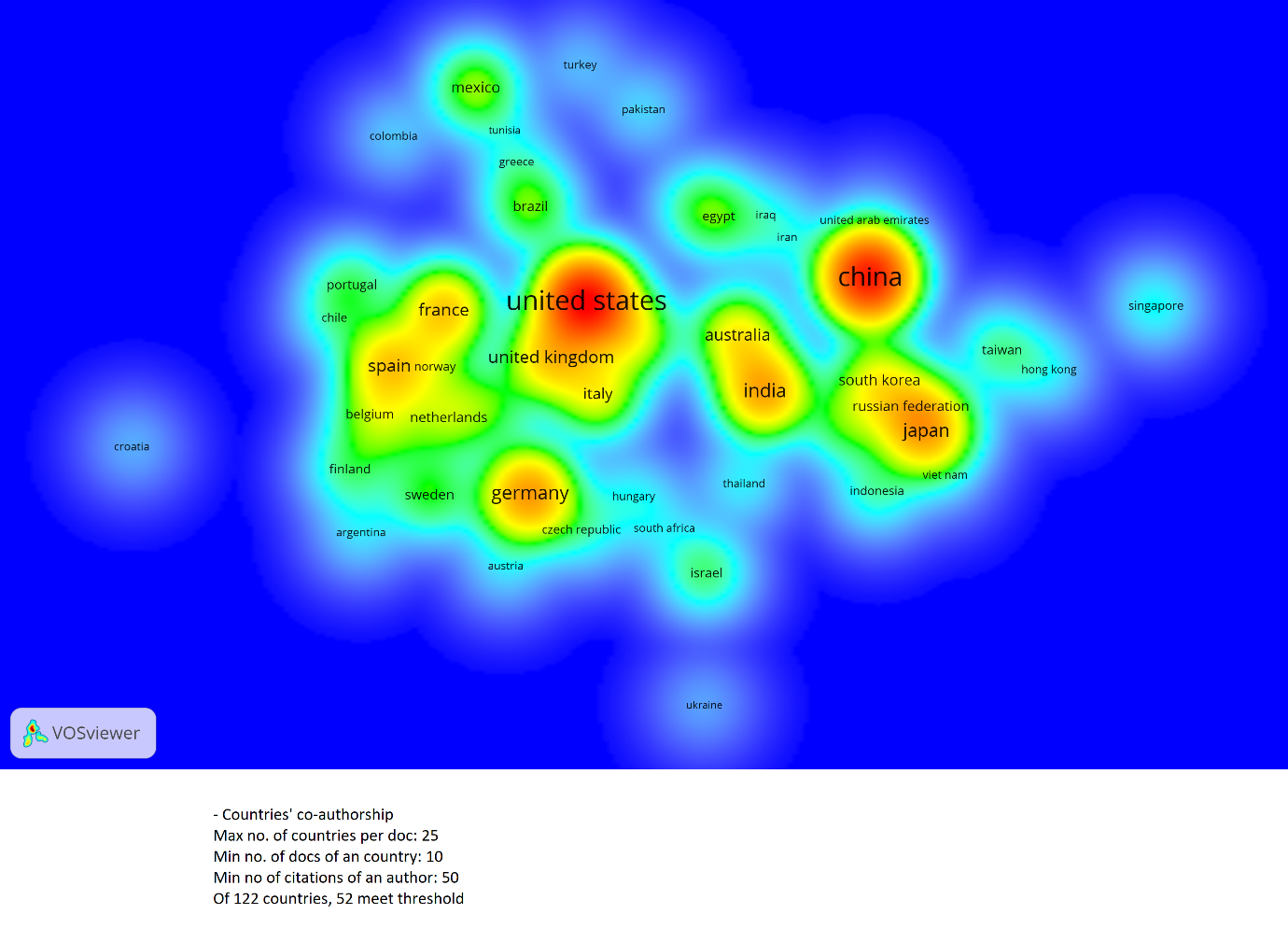


(b)

Figure S3. (a) Network Visualization of countries’ co-authorship (weights: documents). (b) Overlay visualization of countries’ co-authorship in 1937-2023 (weights: documents; scores: average publications per year).
